# Characterization of Putative Virulence Factors of *Pseudomonas aeruginosa* Strain RBS Isolated from a Saltern, Tunisia: Effect of Metal Ion Cofactors on the Structure and the Activity of LasB

**DOI:** 10.1155/2020/6047528

**Published:** 2020-07-23

**Authors:** E. Rigane, R. Dutoit, S. Matthijs, N. Brandt, S. Flahaut, K. S. Belghith

**Affiliations:** ^1^Laboratory of Plant Biotechnology, Faculty of Sciences of Sfax, University of Sfax, Route Sokra BP 1171, 3000 Sfax, Tunisia; ^2^Labiris Research Institute, Avenue Emile Gryzon 1, 1070 Brussels, Belgium; ^3^Laboratory of Applied Microbiology, EIB, Université Libre de Bruxelles, Avenue Emile Gryzon 1, 1070 Brussels, Belgium

## Abstract

*Pseudomonas aeruginosa* is a ubiquitous Gram-negative bacterium able to survive in diverse environments such as soil, plants, freshwater, and seawater. *P. aeruginosa* can be an opportunistic pathogen to humans when their immune system is deficient. Its pathogenicity may be linked to the production of virulence factors. We isolated *P. aeruginosa* strain RBS from the saltern of Sfax in Tunisia. In this study, we characterized the halotolerance, antibiotic susceptibility, and some virulence factors of strain RBS. High NaCl concentrations inhibited growth and motility. However, biofilm formation was enhanced to protect bacteria against salt stress. Among the 18 antibiotics tested, quinolones and tetracycline showed a significant inhibitory effect on growth, motility, and biofilm formation of strain RBS. *β*-Lactams, however, did not have any inhibitory effect on neither bacterial growth nor motility. In some cases, resistance was due, in part, to biofilm formation. We also showed that RBS produces two proteases, LasB and AprA, which have been shown to be implicated in host infection. LasB was further characterized to study the role of metal ions in enzyme stability. It possesses two distinct metal ion-binding sites coordinating a calcium and a zinc ion. The effect of metal ion chelation was evaluated as well as substitutions of residues involved in metal ion binding. Impairing metal ion binding of LasB led to a loss of activity and a sharp decrease of stability. Our findings suggest that the binding of both metal ions is interdependent as the two metal ions' binding sites are linked via a hydrogen bond network.

## 1. Introduction

*Pseudomonas aeruginosa* is a ubiquitous, Gram-negative bacterium that thrives in soil and aquatic environments, playing important roles in the carbon and nitrogen cycles. *P. aeruginosa* is also found infecting plants, insects, fishes, and mammals [1]. During the last few decades, *P. aeruginosa* has become one of the most frequent causative agents of nosocomial infections in predisposed human subjects [2]. *P. aeruginosa* affects a wide category of patients convalescing in hospitals from, for instance, lung diseases, traumatized cornea, and burns. Because of its ability to grow and survive in various environmental conditions, *P. aeruginosa* infection becomes common and outbreaks of extreme drug-resistant strains are frequent among hospital wards and intensive care units [3].

In order to control and prevent these pseudomonal infections, understanding regulatory mechanisms governing virulence gene expression is crucial to develop alternative therapeutic interventions. *P. aeruginosa* produces a variety of virulence factors regulated by quorum sensing (QS): elastase (LasB), alkaline protease (AprA), protease A, exotoxin A, pyocyanin, and rhamnosyltransferase [4]. Recent studies have unveiled a hierarchical QS network in this pathogen, consisting of interconnected signaling pathways [4, 5]. QS contributes to control the production of virulence factors, motility, motility-sessility switch, and biofilm development.

Bacterial motility is a critical aspect of *P. aeruginosa* pathogenesis. Swimming and swarming are the most described forms of motility [6]. These two movements present an essential mechanism for host colonization and biofilm formation [7]. During biofilm formation, *P. aeruginosa* cells attach to surfaces and form microcolonies, embedded in extracellular polymeric substances. Switching to a sessile lifestyle is a survival strategy for *P. aeruginosa* to evade stress and other adverse conditions [5]. The development of a biofilm has been characterized as an essential factor for antimicrobial resistance and tolerance [8]. Consequently, a significant rise in multidrug-resistant (MDR) strains makes the eradication of *P. aeruginosa* difficult [9]. In addition, *P. aeruginosa* utilizes intrinsic and acquired resistance mechanisms to counter the action of most antibiotics [10]. *P. aeruginosa* also produces the blue-green phenazine-derived pigment pyocyanin, involved in a variety of significant biological activities including gene expression and biofilm formation. For this reason, pyocyanin is considered both a virulence factor and a QS signaling molecule [11].

Several extracellular proteases are known to facilitate bacterial colonization by inducing damage to host tissue and actively subverting immune responses [12]. Two of these, AprA and LasB, are often implicated in *P. aeruginosa* infections. AprA degrades flagellin to escape the flagellin-mediated immune response. LasB has a tissue-damage activity by degrading elastin and extracellular matrix components. In addition, it degrades various plasma proteins such as immunoglobulins. Both proteases are zinc metalloproteases that require calcium and zinc ions for stability and catalysis, respectively [13, 14]. Owing to their unique chemical characteristics, metal ions fulfill and carry out diverse biological functions in enzymes. Metal binding is based on a specific geometry coordination imposed by the ligand residues [15]. Therefore, studying the interactions between the metal ions and their ligands is essential to understand their structural and functional properties [15]. Furthermore, how zinc and calcium affect the binding of each other has not been addressed in the literature.

In this study, we isolated a *P. aeruginosa* strain, called RBS, from the saltern of Sfax, Tunisia. The strain RBS was shown to be moderately halotolerant. Despite its isolation from a nonclinical environment, it is resistant to several antibiotics which prompted us to further characterize its virulence factors including pyocyanin, rhamnolipids, and secreted proteases. Our findings allowed the description and characterization of the key strategic adaptation mechanisms including motility, biofilm formation, and antibiotic resistance mechanisms. In the second part of this work, we focused on two secreted proteases, AprA, and LasB. The latter was further characterized to study the role of its metal ion cofactors. According to our results, the two metal ion centers of LasB are important for both the activity and stability. The interdependence of both metal ion centers could be due to a H-bond networking connecting their ligands.

## 2. Material and Methods

### 2.1. Isolation of Strain RBS

Soil samples were collected from the artificial saltern of Sfax located in the southern coast of the Mediterranean Sea (34° 38′ N 10° 42′ E) in Tunisia, to isolate protease-producing bacteria. Therefore, obtained bacterial strains were plated on skim milk agar plates (pH 7), incubated at 30°C, and screened for proteolytic activity after 18 h. The colonies presenting a clear zone, resulting from milk casein hydrolysis, were evaluated as protease producers. For liquid culture, cells were grown for 38 h at 30°C in growth medium (GM) prepared as previously described [15] with slight modifications: 5 g/L of casein supplemented with 10 g/L of wheat bran, at pH 7.3. The supernatant was saturated using ammonium sulfate. All experiments were conducted in triplicate. The strain exhibiting the highest hydrolytic activity on milk casein in solid and liquid conditions was selected and named RBS.

### 2.2. Molecular Identification of Strain RBS

Total genomic DNA of strain RBS was extracted and purified with the QIAamp DNA Mini Kit (Qiagen, Germany). The almost complete 16S rRNA gene sequence (position 29 to 1522 in *E. coli*) of strain RBS was amplified with primers pA and pH [16]. Illumina paired-end sequencing was performed at the Oxford Genomics Center (UK). The 100 bp reads were de novo assembled using Velvet [17] using a minimum contig length cutoff of 1 kb and various *k*-mer lengths. The Whole Genome Shotgun project has been deposited at DDBJ/ENA/GenBank under the accession WMEG00000000. The version described in this paper is version WMEG00000000.1. Annotation of open reading frames (ORF) was performed with the NCBI Prokaryotic Genome Annotation Pipeline (PGAP). Strain RBS was identified at the species level by the type strain genome server (TYGS) of DSMZ (https://tygs.dsmz.de/) [17].

### 2.3. Phenotypic Characterization of *P. aeruginosa* Strain RBS

To study the halotolerance of strain RBS, the growth, motility, and biofilm formation assays were carried out in the presence of NaCl at different concentrations (from 0.2 to 1 M). First, the growth of strain RBS was assessed at 30°C in Luria Bertani (LB) medium with increasing NaCl concentrations. Measurements of OD_600_ were done after 24 h of incubation. Motility assays were performed in LB medium (0.3% agar) [6] and M9 Minimal Medium (M9) (0.6% agar) [18] for swimming and swarming assays, respectively. To assess the influence of NaCl on motility, NaCl was added to a concentration of up to 0.7 M. Twenty mL plates of either LB or M9 were poured and allowed to cool and solidify for, respectively, 20 and 30 min. Subsequently, five *μ*L of an overnight culture of strain RBS at an OD_600_ of 0.4 was deposited at the center of each plate. The plates were then incubated at 30°C for 18 h. Three technical replicates were performed for each condition. The percentage (%) of surface covered in the swarming and swimming assays was measured using ImageJ software, and values were normalized to the control grown in the absence of NaCl.

Biofilm formation was tested as described previously [19]. Cultures were incubated for 24 h at 30°C. The biofilm left adhered to the wells was stained by adding 200 *μ*L of 0.15% (v/v) safranin and incubated for 10 min at room temperature. To solubilize the safranin stain retained by the biofilm, 150 *μ*L of acetic acid 33% (*v*/*v*) was added to each well. The absorbance of the formed biofilm was measured at 531 nm against a blank (acetic acid) and then normalized to biomass (OD_600_). The biofilm development of strain RBS in the presence of NaCl was evaluated in increasing NaCl concentrations (from 0 to 1 M).

The susceptibility of RBS to antibiotics was determined by the disc agar diffusion (DAD) technique according to Bauer et al. [20]. An overnight culture was used to inoculate agar plates (OD_600_ of 0.5). Filter paper discs of 6 mm diameter (GE Healthcare Life Sciences) were used to study bacterial resistance or susceptibility to 18 antibiotics: six *β*-lactam antibiotics (ampicillin, carbenicillin, cephalothin, neomycin, oxacillin, and penicillin), three aminoglycosides (gentamicin, kanamycin, and streptomycin), three quinolones (ciprofloxacin, nalidixic acid, and norfloxacin), vancomycin (glycopeptide), lincomycin (lincosamide class), erythromycin (macrolide), tetracycline (tetracycline class), and two miscellaneous agents (chloramphenicol and trimethoprim). The diameter of the bacterial growth inhibition zone surrounding the disc was accurately measured manually and compared to the European Committee for Antimicrobial Susceptibility Testing (EUCAST) guidelines.

The effect of antibiotics on motility of strain RBS was evaluated in the presence of ampicillin, chloramphenicol, kanamycin and tetracycline (10, 25, and 50 *μ*g/mL), and streptomycin (2.5, 5, and 10 *μ*g/mL). Motility media were autoclaved and placed to cool till 56°C. Antibiotics were then added at the appropriate concentrations. The effect of antibiotics on biofilm formation of the strain RBS was assessed by incorporating different antibiotics at different concentrations in biofilm medium: ampicillin, streptomycin, and tetracycline (5, 10, and 25 *μ*g/mL), kanamycin (25, 50, and 100 *μ*g/mL), and chloramphenicol (5, 10, 25, and 50 *μ*g/mL).

The production of pyocyanin was assayed on Pseudomonas P agar (ThermoFisher Scientific) inoculated with an overnight culture of strain RBS and incubated for 48 h at 30°C. The production and quantification of rhamnolipids (RHL) were carried out according to a methylene-blue-based quantification procedure described previously [21].

### 2.4. Production, Purification, and MS Identification of LasB and AprA

A cell-free supernatant sample was collected from an RBS culture grown for 36 h in LB supplemented with 10 g casamino acids (Difco). Cells were pelleted by centrifuging at 5,500 × *g*. The supernatant was collected and precipitated by adding ammonium sulfate at 65% of saturation. The obtained pellet (centrifuged at 18,000 × *g* for 15 min) was then dialyzed against 20 mM sodium phosphate buffer pH 7.2 (buffer A). The protease purification was carried out by means of an anionic exchange chromatography using a Source 15Q resin (GE Healthcare, Tricorn 10/150 column). The column was equilibrated with buffer A prior to the purification. The elution was performed using a gradient step from 0 to 1 M NaCl in a ten-column volume. Fractions were analyzed by SDS-PAGE and caseinolytic activity assay [22]. Fractions containing proteolytic activity were pooled and concentrated using Amicon ultrafiltration units of 10 kDa cutoff (Merck Millipore). Produced proteases were identified by LC-ESI-MS/MS analysis from a Coomassie Blue-stained gel (GIGA Proteomics Facility, University of Liège, Belgium). Zymogram analysis was performed according to Abcam's protocols.

### 2.5. Cloning of *lasB*

A codon-optimized synthetic gene (GeneArt—ThermoFisher Scientific) was used for the heterologous expression of *lasB* in *Escherichia coli*. The gene was amplified by PCR using specific primers (5′-ATTATTCATATGAAATACCTGCTGCCGACCGCTGCTGCTGGTCTGCTGCTCCTCGCTGCCCAGCCGGCGATGGCCGCCGAAGCCGGTGGTCCTGG-3 ′and 5 ′-AATAATCTCGAGTTACAGTGCGCTCGGACAGGTAACA-3′) using Pfu DNA-polymerase (ThermoFisher Scientific). The amplified PCR product was cloned in the pJET1.2/blunt vector (ThermoFisher Scientific) prior to sequencing. The *lasB* gene was then excised by digesting with *Nde*I and *Xho*I and cloned into the pET30b expression vector (Novagen) using T4 DNA ligase (ThermoFisher Scientific). The plasmid sequences were verified by sequencing (Genetic Service Facility, University of Antwerp). In order to examine the role of calcium and zinc ions in catalysis and stability, substitutions of ion ligands in alanine and/or isomeric amino acids were carried out using synthetic genes (GeneArt, ThermoFisher Scientific). The mutated genes were subcloned into pET30b expression vector. The *E. coli* strain MC1061 was used for gene cloning.

### 2.6. Production and Purification of Recombinant Proteases

The *E. coli* strain BL21 was used for recombinant production of LasB. Cells were grown in 1.5 L of ZYM broth [23] containing 50 *μ*g/mL kanamycin. After 24 h of incubation, both culture supernatant and cell pellet were collected by centrifuging at 9,000 × *g*, 4°C for 30 min. The cell pellet was washed twice with 50 mM potassium phosphate buffer pH 6.0 and then resuspended in the same buffer. The periplasmic fraction was prepared following the protocol developed by Piwpankaew et al. [24] with slight modifications. Cells were resuspended in 100 mM MOPS and 0.5 M sucrose, pH 8. After incubating for 5 min on ice, cells were pelleted by centrifuging at 9,000 × *g* for 15 min and resuspended in 1 mM MgCl_2_. The supernatant was incubated for 5 min on ice and then collected by centrifugation and neutralized with 40 mM MOPS buffer supplemented with EDTA-free Protease Inhibitor (Roche Applied Science).

### 2.7. Protease Activity Assays

Four DQ-substrates (ThermoFisher Scientific) were used to determine the specific activity of purified proteases against different natural substrates: DQ-gelatin (from pig skin), DQ-collagen type I (from bovine skin), DQ-collagen type IV (from human placenta), and DQ-elastin (from bovine neck ligament). Activity assays were carried out in a black 96-well microplate (Corning) according to the manufacturer's recommendations: 0.7 *μ*g/mL of enzyme was incubated in the presence of 100 *μ*g/mL DQ-gelatin, 100 *μ*g/mL DQ-collagen types I and IV, and 25 *μ*g/mL DQ-elastin diluted in 1X reaction buffer. This buffer is composed of 0.5 M Tris-HCl, 1.5 M NaCl, 50 mM CaCl_2_, and 2 mM sodium azide, pH 7.6 (for DQ-gelatin and DQ-collagen types I and IV), and 1 M Tris-HCl and 2 mM sodium azide, pH 8.0 (for DQ-elastin), in a reaction volume of 80 *μ*L. Fluorescence was measured every 30 s for 20 min at 40°C on a Spectra Max M5 Multi-Mode Microplate Reader (Molecular Devices) with excitation and emission wavelength set at 495 nm and 515 nm, respectively. The data were corrected by subtracting the negative control fluorescence. A collagenase type IV from *Clostridium histolyticum* was used as positive control for assays with DQ-gelatin and DQ-collagen types I and IV while an elastase from *Sus scrofa* pancreas served as a positive control with DQ-elastin as substrate. All measurements were carried on triplicate. The maximal velocity (*V*_max_) and Michaelis constant (*K*_m_) were determined for DQ-gelatin and DQ-elastin as substrate using GraphPad Prism 6.01.

For determining the optimum pH of the enzyme, the protease activity was performed by using glycine-HCl (pH 5.0), sodium phosphate (pH 6.0-8.0), Tris-HCl (pH 8.0-9.0), glycine-NaOH (pH 9.0-11.0), and Na_2_HPO_4_-NaOH (pH 11) buffer at 0.1 M containing 1% casein. The enzyme was incubated in various buffer solutions (pH 3-13) at room temperature for 2 h, and the residual activity was determined in standard experimental conditions. The effect of temperature on the enzyme activity was determined by measuring the enzyme activity on 1% casein in phosphate buffer (40 mM, pH 8.0) for 10 min at different temperatures, ranging from 30°C to 80°C. The nonheated enzyme was considered the control (100% activity).

The susceptibility of LasB to inhibitors was determined using phenylmethylsulfonyl fluoride (PMSF), bestatin, ethylenediaminetetraacetic acid (EDTA), ethylene glycol-bis (*β*-aminoethyl ether)-N,N,N′,N′-tetraacetic acid (EGTA), 1, 10-phenanthroline, diethylenetriaminepentaacetic acid (DTPA), and *β*-mercaptoethanol. The residual enzymatic activity was measured after incubating 25 *μ*M LasB with two doses of inhibitors, 1 and 5 mM, at 30°C for 30 min. The effect of metal ion on LasB activity was investigated by incubating LasB (25 *μ*M) with two doses, 1 mM and 5 mM, of CaCl_2_, CoCl_2_, and ZnSO_4_ at room temperature for 30 min. The activity of LasB without any treatment was taken as 100%.

### 2.8. Thermal Shift Assay (TSA)

LasB was diluted to 30 *μ*M in 20 mM MOPS pH 7.2, Sypro Orange 5X (ThermoFisher Scientific) with or without EDTA, EGTA, and 1,10-phenanthroline (300 *μ*M), as described previously [25]. The samples were distributed in a PCR multistrip (20 *μ*L in each well). TSA was carried out using a StepOnePlus Real-Time PCR System (ThermoFisher Scientific) with a temperature gradient from 15°C to 99°C at +0.4°C/min ramp. All experiments were performed in duplicate. The wavelengths for excitation and emission were 490 and 575 nm, respectively. The derivative of the fluorescence curve was used to determine the melting temperature (*T*_m_).

### 2.9. Statistical Analysis

The Graphpad Prism software (version 6.01) was used for statistical analysis. Each experiment was performed at least 3 times, and the data were calculated as means ± SD (standard deviation). One-way parametric ANOVA was performed to compare means, in accordance with the Brown-Forsythe law and Holm-Sidak test.

## 3. Results

### 3.1. Isolation of the Strain RBS and Characterization of Its Halotolerance

**Figure 1:**
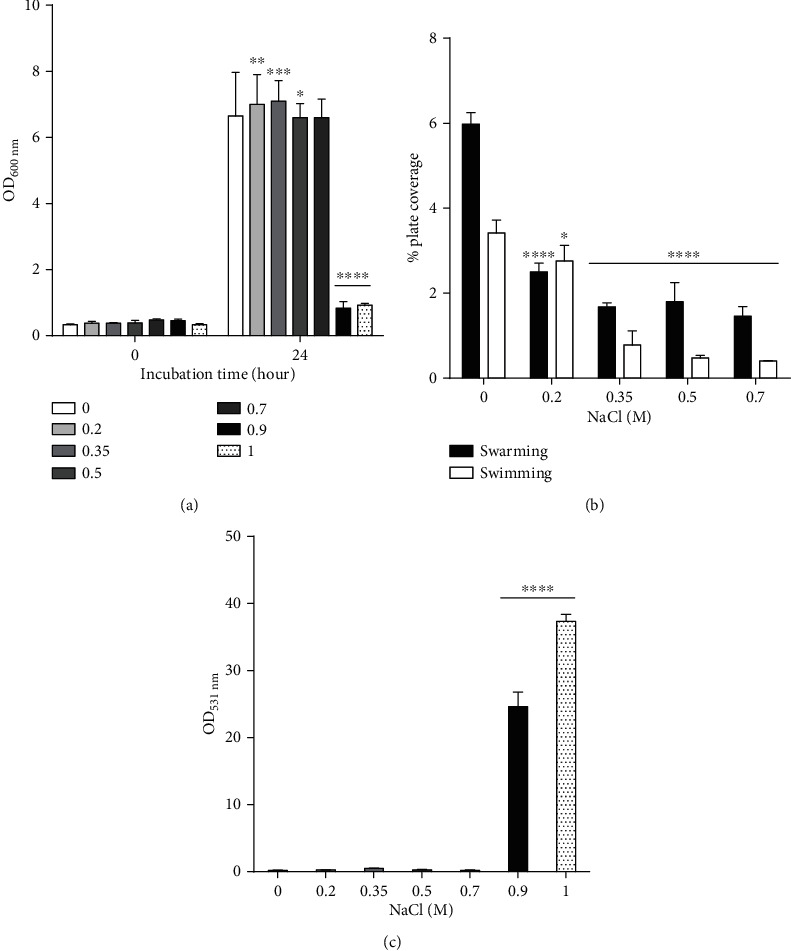
Effect of sodium chloride (NaCl) on *P. aeruginosa* strain RBS (a) growth, (b) motility, and (c) biofilm formation. Various concentrations of NaCl ranging from 0 to 1 M were tested to monitor its effect on growth and biofilm formation. For motility assays, NaCl was added at a concentration ranging from 0 to 0.7 M in solid growth medium. The data of motility assays are expressed as plate coverage percentage. For biofilm formation, OD_531_ was normalized to biomass OD_600_. Values are expressed as means ± SD. ^∗^*P* < 0.05, ^∗∗^*P* < 0.01, ^∗∗∗^*P* < 0.001, and ^∗∗∗∗^*P* < 0.0001 vs. control.

Samples were collected from an artificial saltern of Sfax, Tunisia, in April 2015. Three proteolytic strains were isolated from the saltern pond (90.7 ± 19.1 psu). Strain RBS showed the largest clear hydrolysis zone using casein as substrate (see Figure S1 in the Supplementary Materials for casein hydrolysis zone), with the highest specific activity (1773.45 U/g of protein). Its halotolerance was evaluated by growing cells in LB medium with increasing NaCl concentrations. Biomass was significantly enhanced at concentrations of up to 0.7 M NaCl, compared to growth in the absence of salt (*P* < 0.0001) (Figure 1(a)). At these concentrations, the swarming state of cells was more important than the swimming state (Figure 1(b)). At higher concentrations (0.9 and 1 M), biomass and motility decreased significantly (*P* < 0.0001), while biofilm formation was significantly enhanced compared to biofilm in the absence of NaCl (*P* < 0.0001) (Figure 1(c)). These results showed the shift of strain RBS population from swimming to swarming (movement in a group) and the subsequent formation of microcolonies embedded in the biofilm matrix.

### 3.2. Molecular Identification of Strain RBS

The genome of RBS has a total size of 6.66 Mbp with 5,696 genes, of which 5,584 are coding genes. Blast analysis of the 16S rRNA gene (MT031760) identified strain RBS as *P. aeruginosa*. Analysis of the genome at the type strain genome server (TYGS) confirmed the species identification.

### 3.3. Antibiotic Resistance of *P. aeruginosa* RBS

The *P. aeruginosa* strain RBS displayed a broad-range resistance to antibiotics, notably against *β*-lactam antibiotics, vancomycin, trimethoprim, and erythromycin (Table 1). Gentamycin, kanamycin, and streptomycin were the most effective antibiotics against strain RBS with a growth inhibition zone of 20, 15, and 12 mm diameter, respectively. For the quinolones, an overall notable sensitivity was recorded against nalidixic acid (7 mm) and ciprofloxacin (15 mm). The sensitivity to quinolones is due in part to the absence of single nucleotide mutation in *gyrA* (codon 83, T83I), *parC* (codon 87, S87L), and *mexR* (codon 126, E126V), coding the DNA gyrase subunit A, topoisomerase IV, and MexAB-OprM pump, respectively. The genome sequence of strain RBS does not show any mutation in *gyrA* and *parC*, but *mexR* displays the mutation of codon 126. This mutation, however, is not thought to be linked to quinolone resistance as reported previously [26].

### 3.4. Effect of Antibiotics on Motility and Biofilm Formation of *P. aeruginosa* RBS

**Figure 2:**
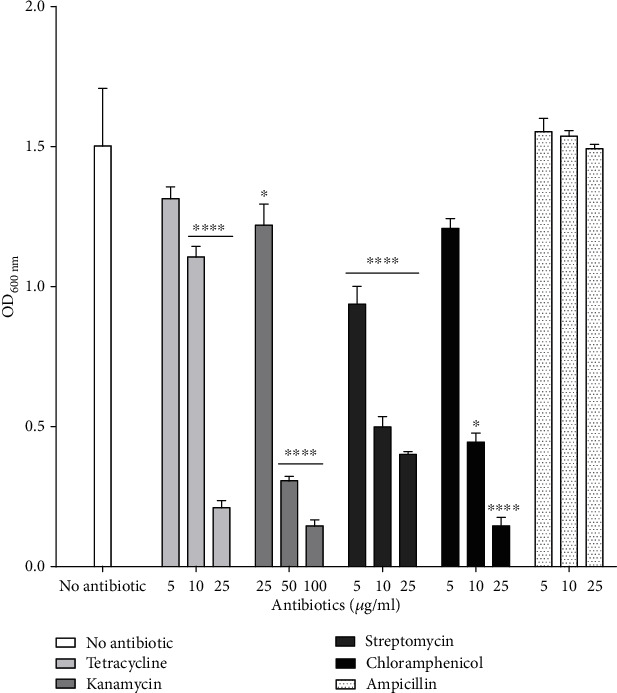
Influence of antibiotics on *P. aeruginosa* RBS growth. Tetracycline, kanamycin, chloramphenicol, streptomycin, and ampicillin were added to growth media at different concentrations. Growth was determined by following OD_600_ and normalized to strain RBS grown without antibiotic. Values are expressed as means ± SD. ^∗^*P* < 0.05, ^∗∗^*P* < 0.01, ^∗∗∗^*P* < 0.001, and ^∗∗∗∗^*P* < 0.0001 vs. control with *n* = 3.

High doses of chloramphenicol, tetracycline, and streptomycin (25 *μ*g/mL) and kanamycin (50 *μ*g/mL) significantly inhibited *P. aeruginosa* RBS growth in liquid medium (Figure 2). Ampicillin had no effect on cell growth, even at a concentration of 25 *μ*g/mL. This resistance can probably be attributed to the production of *β*-lactamases, since the strain RBS genome contains *β*-lactamases coding genes.

**Figure 3:**
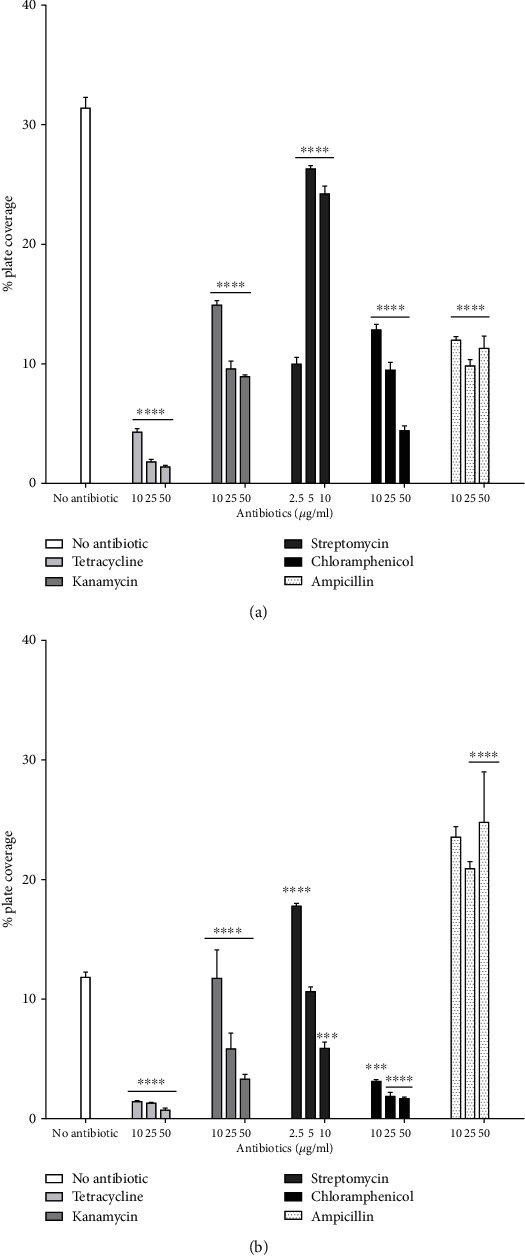
Effect of antibiotics on (a) swimming and (b) swarming of *P. aeruginosa* RBS cells. Tetracycline, kanamycin, chloramphenicol, streptomycin, and ampicillin were added at different concentrations to swimming and swarming agar media to determine their effect on motility. Growth was normalized to the control grown without antibiotic. Data are expressed as plate coverage percentage. Values are expressed as means ± SD. ^∗∗∗^*P* < 0.001, ^∗∗∗∗^*P* < 0.0001 vs. control grown without antibiotic with *n* = 3.

Regarding its motility, *P. aeruginosa* strain RBS had a differential response to antibiotic exposure. Swimming and swarming motility was dramatically hampered by tetracycline and chloramphenicol at a concentration ranging from 10 *μ*g/mL to 50 *μ*g/mL, (*P* < 0.0001) (Figures 3(a) and 3(b)). Kanamycin significantly inhibited swimming motility at a low concentration (10 *μ*g/mL), (*P* < 0.0001) (Figure 3(a)). The swarming phenotype, however, was reduced at a higher concentration of kanamycin (Figure 3(b)). In the presence of streptomycin (2.5 *μ*g/mL), swarming was significantly enhanced (*P* < 0.0001), (Figure 3(b)), while in higher concentrations, RBS cells shifted to swimming state to overcome the presence of high streptomycin concentrations (Figure 3(a)). When *P. aeruginosa* RBS was exposed to ampicillin, the swimming motility was decreased while the swarming motility was significantly increased by ampicillin, from 11.81% of plate coverage without antibiotic to 24.78% with 50 *μ*g/mL ampicillin.

**Figure 4:**
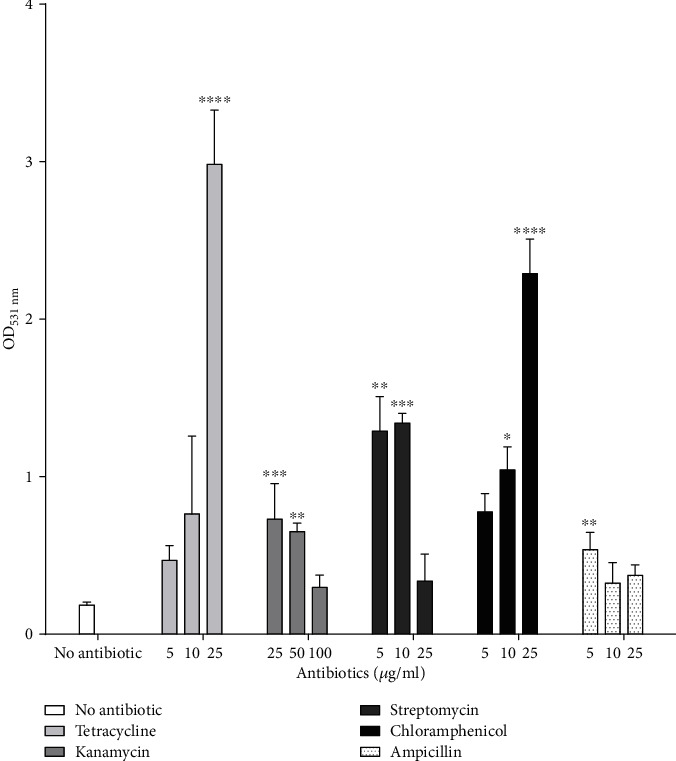
Influence of antibiotics on *P. aeruginosa* RBS biofilm formation. Tetracycline, kanamycin, chloramphenicol, streptomycin, and ampicillin were added at different concentrations to biofilm medium. Data are expressed as OD_531_ and normalized to OD_600_. Values are expressed as means ± SD. ^∗^*P* < 0.05, ^∗∗^*P* < 0.01, ^∗∗∗^*P* < 0.001, and ^∗∗∗∗^*P* < 0.0001 vs. control with *n* = 3.

The five tested antibiotics, however, promoted biofilm formation in *P. aeruginosa* strain RBS (Figure 4) compared to the control culture without antibiotic. Biofilm formation was especially high in the presence of 100 *μ*g/mL of chloramphenicol (OD_531_ = 6.3). Nevertheless, biofilm formation of *P. aeruginosa* strain RBS was inhibited at the highest concentration of kanamycin (100 *μ*g/mL) and streptomycin (25 *μ*g/mL) (Figure 4). Enhanced biofilm production is a common strategy to tolerate antibiotics in *P. aeruginosa* as the extracellular matrix provides a reduced permeability to antibiotics [27]. Under the exposure to antibiotics, RBS cells shift from a free state (swimming and swarming) to a sessile state to overcome the exposure to antibiotics. Indeed, efflux systems are upregulated in biofilms, increasing tolerance to antibiotics [28].

### 3.5. Virulence Factors of *P. aeruginosa* Strain RBS

*P. aeruginosa* secretes various redox-active phenazine compounds, the most well-studied being pyocyanin. The pyocyanin production was evaluated on Pseudomonas P agar medium. After 48 h, a green pigment diffused into the agar, indicating the production of pyocyanin (see Figure S2 in the Supplementary Materials for pyocyanin production). The production of RHL in *P. aeruginosa* strain RBS was assessed using the methylene blue complexation method. Under these conditions (M9 medium, at 30°C for 24 h), about 94 *μ*g of RHL was produced per milliliter of culture. The production of rhamnolipids is associated with the expression of pyocyanin. The production of proteases is a well-known virulence trait of *P. aeruginosa* [29]. As RBS displayed a strong hydrolytic activity on casein (see Figure S1 in the Supplementary Materials for casein hydrolysis zone), the secreted proteases were identified by SDS-PAGE and LC-ESI-MS/MS. On SDS-PAGE gel, two major bands were observed at 33 kDa (elastase, LasB) and 50 kDa (alkaline protease, AprA) (see Figure S3-A in the Supplementary Materials for SDS-PAGE gel) that has been determined by LC-ESI-MS/MS, with pI 6.3 and 4.1, respectively. Based on their pI, an anionic chromatography was used to separate LasB and AprA, at pH 7.2. AprA was eluted at 0.65 and 0.75 M NaCl while LasB was not retained on the chromatography media. The proteolytic activity of both enzymes was assessed by casein hydrolysis assay and zymogram (see Figure S3-B in the Supplementary Materials for zymogram). Specific activity of purified LasB and AprA against casein was 6843 and 828.5 IU/mg, respectively.

### 3.6. Biochemical Characterization of Recombinant LasB

**Figure 5:**
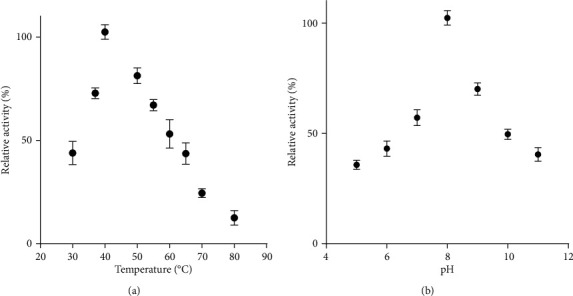
Influence of temperature (a) and pH (b) on the activity of recombinant LasB. The temperature profile was determined by assaying enzyme activity at different temperature values ranging from 30 to 80°C. The pH optimum profile was determined at 40°C in different buffers. Error bars represent SD with *n* = 3.

LasB and AprA were produced recombinantly in *E. coli* for further characterization. Unfortunately, AprA failed to be secreted and was present in the insoluble cell fraction only (data not shown). For LasB, the periplasmic translocation was mediated via the secretion signal of PelB [30], making the purification of recombinant LasB possible from the periplasm by anionic chromatography. LasB displayed a proteolytic activity up to 80°C, with a maximum at 40°C (Figure 5(a)). A sharp decrease was observed from about 65°C. LasB activity was assayed in a pH range from 4 to 11 and exhibited an optimum at pH 8 (Figure 5(b)).

The specificity of LasB was evaluated against four fluorescent-labelled substrates: DQ-elastin, DQ-collagen of types I and IV, and DQ-gelatin (Table 2). LasB displayed the highest specificity on gelatin substrate, which was expected as gelatin is the product of collagen denaturation. With the native substrates, LasB is more specific to elastin degradation than type I and IV collagens. Kinetic parameters, *k*_cat_ and *K*_m_, were determined with DQ-elastin and DQ-gelatin as substrates. The hydrolysis rate of both substrates obeyed the Michaelis-Menten equation (see Figure S4 in the Supplementary Materials for Michaelis-Menten curve). Only apparent kinetic constants were determined due to the substrate solubility limitation. For DQ-gelatin degradation (pH 8.0, 40°C), *K*_m_ and *k*_cat_ were 95 *μ*g/mL and 588 s^−1^, respectively, while being 92 *μ*g/mL and 0.17 s^−1^ for DQ-elastin hydrolysis.

### 3.7. The Role of Metal Ion Cofactors on LasB Activity and Stability

Several known protease inhibitors (*β*-mercaptoethanol, DTPA, EDTA, EGTA, 1,10-phenanthroline, and PMSF) were tested on LasB (Table 3). The activity remained unaffected by PMSF, which is consistent with the absence of a catalytic serine residue. *β*-Mercaptoethanol increased slightly the activity, which may be due to the enzyme stabilization in the tested conditions. In the presence of EDTA and 1,10-phenanthroline at 5 mM, LasB retained only 56.2 and 26.0% of activity, respectively. The two other metal-chelating inhibitors, DTPA and EGTA, had an even more dramatic impact on elastase activity, being reduced to 11.5% and 3.4%, respectively. Similar results were reported for AMPP, an elastase from *P. aeruginosa* [31]. Two variants affected in Zn^2+^ or Ca^2+^ binding were generated to evaluate the contribution of both ions to the LasB activity and stability. To impair the Zn^2+^ binding, His-140 and His-144 were substituted with an alanine residue (variant LasB_Zn^2+^). For the Ca^2+^ binding, Asp-136, Glu-172, Glu-175, and Asp-183 were substituted with asparagine and glutamine residues (mutant LasB_Ca^2+^). After the purification of the two variants from *E. coli* periplasm, the enzymatic activities were assessed using DQ-gelatin as substrate. Both variants were inactive, suggesting that Ca^2+^ and Zn^2+^ could play an important role in the LasB activity. The contribution of Ca^2+^ and Zn^2+^ to the enzyme stability was determined by thermal shift assays using either the wild-type enzyme in presence of chelating agents or the metal-binding impaired variants.

Without any chelating agent, LasB had a *T*_m_ of 77.0°C. In the presence of EDTA and 1,10-phenanthroline, a zinc specific chelator, the *T*_m_ values dropped to 54.8°C and 54.4°C, respectively (Table 4). With EGTA, a calcium specific chelator, LasB had a *T*_m_ of 66.0°C. The chelation of Ca^2+^ and Zn^2+^ could lead to a less stable “open” conformation of LasB, explaining the *T*_m_ drop in the presence of the chelator. As 1,10-phenanthroline had a greater impact on LasB stability than EGTA, one may suppose that Zn^2+^ is important for the stability in addition to its catalytic role. For mutants LasB_Ca^2+^ and LasB_Zn^2+^, measured *T*_m_ were similar, 47.4°C and 48.6°C, respectively. This result suggests that both Ca^2+^- and Zn^2+^- binding sites are implicated in structure integrity. Based on TSA data, the binding of metal ions in LasB made the structure more stable, suggesting a possible reorganization of the active site pocket.

**Figure 6:**
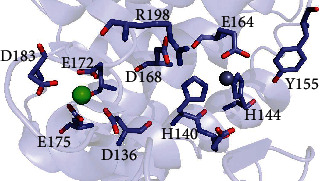
Close-up view into the active site of elastase (PDB 1U4G). Selected residues are displayed in stick representation, the calcium ion as a green sphere, and the zinc ion as a grey sphere.

According to our results, impairing calcium or zinc binding had an impact on the LasB activity and stability. Based on LasB structure (PDB code 1U4G), both metal ion-binding sites are intertwined by a hydrogen bond network. The interactions between residues of the two binding sites were calculated using PDB2PQR server [32]. His-140 and His-144 are in interaction with the neighbor amino acids Glu-141, Tyr-155, Glu-164, and Asp-168. Glu-141 is the catalytic base, Tyr-155 is involved in substrate binding, and Glu-164 is the third Zn^2+^ ligand (Figure 6). Asp-168 is not directly involved in the catalytic site but it interacts with Arg-198 which is involved in substrate binding. The zinc-binding site is connected to the calcium-binding site via interactions involving Asp-136 and Glu-172. Indeed, there are two backbone-to-backbone interactions between Asp-136 and His-140 and between Glu-172 and Asp-168.

## 4. Discussion

The isolation of *P. aeruginosa* strain RBS from the saltern of Sfax raises questions about its origin. Indeed, the high metabolic versatility of *P. aeruginosa* allows its survival in a wide range of environments, including freshwater, wastewater, ocean, and soil. A human-related origin, however, cannot be excluded since *P. aeruginosa* has been found in sewage contaminated rivers [29]. Its occurrence in freshwater is often related to human activities, and therefore, a contamination of coastal water could be possible as shown previously [33]. Although the majority of epidemiological studies have focused on clinical rather than environmental isolates and research into the environmental distribution of strains is still limited [34], Rutherford et al. studied the prevalence of two genes coding effector proteins, *exo*S and *exo*U, in the environment. The *exo*S gene is often found in strains isolated from natural environmental sites (e.g., plant, soil, and lakes), whereas *exo*U occurs in strains isolated from human activity-related sites (e.g., drains and sinks). The genome of strain RBS harbored only *exo*S, suggesting its natural environmental origin [34].

Firstly, we evaluated the growth of the strain RBS in the presence of NaCl. Its growth was enhanced in the presence of up to 0.7 M of NaCl (41 g/L), a concentration close to seawater salinity (40 g/L) [35]. A loss of growth, however, was observed at higher salt concentrations (0.9 and 1 M NaCl). This observation suggests that strain RBS is slightly halophilic. We assessed the effect of NaCl on the *P. aeruginosa* RBS motility. A sharp decrease of swimming motility was observed for NaCl concentrations above 0.5 M. At 0.7 M NaCl, most of the RBS population shifted to swarming movement. At a salt concentration of 0.9 and 1 M, the growth and motility were dramatically reduced, corroborating with biofilm formation. The shift from planktonic state to sessile lifestyle (biofilm) is also detected in some clinical strains as a strategy to escape some stresses, like antibiotic treatment during infection [36, 37]. For this reason, we evaluated the susceptibility to antibiotics, in addition to halotolerance and motility, for *P. aeruginosa* RBS, as clues on its possible environmental origin.

Secondly, the antibiotic susceptibility of the strain RBS was tested against 18 antibiotics. The strain RBS showed susceptibility to aminoglycosides, chloramphenicol, ciprofloxacin, streptomycin, and tetracycline, but it is resistant to *β*-lactam antibiotics, vancomycin, trimethoprim, and erythromycin. Such an antibiotic response pattern has been reported for other marine *Pseudomonas* strains [38]. However, in the presence of kanamycin, ampicillin, and streptomycin, strain RBS displayed the same stress response: biofilm formation. The strain RBS displayed some physiological adaptations, including biofilm formation, to cope with high osmolality and antibiotics. Biofilm can be regarded as a protective hydrated microenvironment to sessile *P. aeruginosa* exposed to desiccation stress [39]. This intrinsic resistance mechanism ensures the survival of biofilm-embedded cells, by reducing the diffusion of antibiotics or by the production of antibiotic degrading enzymes [40]. The phenotypic switch from a free-swimming planktonic lifestyle to biofilm sessility is a common survival strategy of *P. aeruginosa* even though it lowers its virulence [5].

The ability of *P. aeruginosa* to resist antibiotics and environmental stresses makes the management of such infections a huge challenge in the healthcare settings. The pathogenic potential of *P. aeruginosa*, however, is not restricted to its metabolic/genetic versatility or its intrinsic/acquired antibiotic resistance. The production of a powerful arsenal of virulence factors (pigments, pili, flagellum, rhamnolipids, and hydrolytic enzymes) makes *P. aeruginosa* infections highly invasive and toxigenic. Consequently, the development of new therapeutic strategies against multidrug-resistant *P. aeruginosa* is urgently needed. These strategies, however, should not affect the microbial growth as resistant subpopulations may appear during persistent infections [41]. Therefore, the attenuation or inhibition of these virulence factors emerges as an alternative and promising antivirulence therapy to overcome antibiotic-refractory infections caused by *P. aeruginosa*. Bacterial motility inhibition may be also an important therapy target, in order to avoid *P. aeruginosa* cell adherence to host tissues like the lungs. Elastase outstands as a pivotal virulence attribute during the infectious process, playing multifunctional roles in different aspects of the pathogen-host interaction and swarming motility [42]. Previous studies showed that inhibiting the expression/activity of bacterial virulence factors contributes to disarm the *P. aeruginosa* infective arsenal [41]. The virulence quenching by antivirulence compounds can allow the host immune system to prevent efficient bacterial colonization as well as to extinguish an established infection [43].

Finally, we characterized the elastase produced by the strain RBS. Since elastase is a metallopeptidase, it is important to understand and elucidate the roles of divalent metal ions (calcium and zinc) in the elastase stability and activity. For further biochemical characterization, LasB was recombinantly produced in *E. coli*. The effect of temperature and pH on the activity was studied as well as its substrate specificity. LasB displayed a maximal hydrolytic activity at 40°C and was active in a broad pH range (from 4 to 11). It hydrolyzed preferentially gelatin and elastin. The degradation of collagen is slower than that of gelatin and elastin since collagen is a denatured protein substrate. The catalytic activity of LasB depends on a zinc ion located in the active site. LasB possesses a second metal ion-binding site bridging a calcium ion. The role of the calcium ion could be linked to the stability of LasB [44]. Nevertheless, to our knowledge, there is no available study systematically investigating the effect of specific changes in the metal-containing active site (of both calcium and zinc) on the function of LasB. In this work, we addressed this issue by either using chelating agents or substituting the ligands of the calcium- and zinc-binding sites. In the presence of metallic chelators, EDTA, EGTA, and 1,10-phenanthroline, the activity and the thermal stability were extensively affected, suggesting that both Zn^2+^ and Ca^2+^ are involved in activity and stability. The mutation of calcium ligands led to the loss of proteolytic activity as well as to a drop of melting temperature. The same behavior was observed for the LasB_Zn^2+^ mutant. These results suggest that both Ca^2+^ and Zn^2+^ are catalytically and structurally important or both metal ion-binding sites are interconnected.

According to the structure of LasB [13], a hydrogen bond network could link both metal ion-binding sites. The ligands involved in zinc binding are interconnected by hydrogen bonds to other important residues. His-140 interacts with His-223, a residue implicated in substrate binding. Glu-164 establishes hydrogen bonds with Tyr-155, Asp-168, Glu-141, and Arg-198. Tyr-155 and Arg-198 are also involved in substrate binding while Glu-141 is the catalytic acid/base residue. His-140 is also connected to Arg-198 via its interaction with Asp-168. The Zn^2+^-binding site is probably connected to the Ca^2+^-binding site through two backbone-to-backbone interactions involving Asp-136/His-140 and Glu-172/Asp-168. The zinc ion could also play a switcher role via the residues involved in its binding interacting with neighbor amino acids, like Glu-141, Asp-168, and Tyr-155. As previously reported for the zymogen form of matrix metalloproteinase-9 [45], the zinc ion has a structural function and a catalytic role. During the maturation of this protease, one of the ligands can release its interaction with the zinc ion, changing from a tetracoordination to a pentacoordination geometry. Such a coordination geometry swap turns the zinc ion function from a structural role to a catalytic role. To understand how metal ion may affect LasB structure, the apoform and transition state intermediates must be identified and studied.

## 5. Conclusion

The *P. aeruginosa* strain RBS was isolated from the saltern of Sfax in Tunisia. RBS is adapted to its saline environment but is also resistant to many antibiotics and produces several virulence factors (pyocyanin, rhamnolipids, LasB, and AprA). Consequently, the problem arises of how to deal with potential pathogen in the clinical environment. These factors could potentially participate in host infection and host immune component degradation. Their expression, however, depends on a pool of factors through the QS network [4]. But how the expression is induced remains elusive. We investigated the effect of metal ion cofactors (calcium and zinc ions) on the structure and the activity of LasB, a potential target for antivirulence therapies against *P. aeruginosa* infections. Our results suggest that both calcium and zinc ions could play a role in structure stability and activity. Based on available structural data, the two metal ion-binding sites seem to be connected via a hydrogen bond network involving several interrelated conserved residues.

## Figures and Tables

**Table 1:** Susceptibility of *Pseudomonas aeruginosa* strain RBS to various antibiotics determined using disc agar diffusion method.

Antibiotic class	Antibiotic	Disc content (*μ*g)^a^	Zone diameter (mm)
*β*-Lactam antibiotics	Carbenicillin	100	0
	Penicillin	10	0
	Ampicillin	10	0
	Cephalothin	30	0
	Oxacillin	1	0
	Neomycin	30	11

Aminoglycosides	Streptomycin	10	12
	Kanamycin	50	15
	Gentamycin	10	20

Quinolones	Norfloxacin	10	0
	Nalidixic acid	30	7
	Ciprofloxacin	25	15

Tetracycline	Tetracycline	50	13
Lincosamide	Lincomycin	10	0
Glycopeptide	Vancomycin	30	0
Miscellaneous agents	Trimethoprim	50	0
	Chloramphenicol	10	0
Macrolide	Erythromycin	15	0

^a^Except penicillin (U).

**Table 2:** Specific activity of LasB against DQ-elastin, DQ-gelatin, and DQ-collagen of types I and IV. Specific activity is expressed in fluorescence unit (FU) per enzyme concentration (nM) and second (s).

DQ-substrate	Specific activity (FU nM^−1^ s^−1^)
DQ-elastin	0.13
DQ-collagen type I	0.041
DQ-collagen type IV	0.025
DQ-gelatin	0.3

**Table 3:** Influence of inhibitors on proteolytic activity of LasB. The tested inhibitors (1 and 5 mM) were phenylmethylsulfonyl fluoride (PMSF), ethylenediaminetetraacetic acid (EDTA), ethylene glycol-bis(*β*-aminoethyl ether)-N,N,N′,N′-tetraacetic acid (EGTA), 1,10-phenanthroline, diethylenetriaminepentaacetic acid 382 (DTPA), and *β*-mercaptoethanol. The activity is expressed as relative activity with the enzyme incubated without an inhibitor as 100% reference. Values are expressed as means ± SD, *n* = 3.

Inhibitor	Relative activity (%) with 1 mM inhibitor	Relative activity (%) with 5 mM inhibitor
*β*-Mercaptoethanol	117.0 ± 1.2	111.4 ± 1.1
DTPA	45.7 ± 0.6	11.5 ± 1.8
EDTA	84.20 ± 0.6	56.2 ± 0.7
EGTA	82.6 ± 1.0	3.4 ± 0.4
1,10-Phenanthroline	33.9 ± 1.0	26.0 ± 0.6
PMSF	91.3 ± 2.7	121.5 ± 1.6

**Table 4:** Thermal shift assay of inhibitor effect on LasB thermostability. Thermostability was determined by measuring the melting temperature (*T*_m_). The tested inhibitors were ethylenediaminetetraacetic acid (EDTA), ethylene glycol-bis(*β*-aminoethyl ether)-N,N,N′,N′-tetraacetic acid (EGTA), and 1,10-phenanthroline. Values are expressed as means ± SD.

Inhibitor	*T*_m_ (°C)
No chelating agent	77.0 ± 0.1
EDTA	54.8 ± 0.3
EGTA	66.0 ± 0.2
1,10-Phenanthroline	54.4 ± 0.1

## Data Availability

All data are available in the manuscript and genomic database (for genomic sequence).

## Supplementary Materials

Supplementary Materials Figure S1: casein hydrolysis. Figure S2: pyocyanin production by *P. aeruginosa* RBS on Pseudomonas agar P medium. Figure S3: SDS-PAGE and zymography assays of the bacterial lysate from *P. aeruginosa* strain RBS. Figure S4: initial velocity as a function of DQ-substrates (*μ*g/mL).
