# Supplementary material for: Characterization of Putative Virulence Factors of Pseudomonas aeruginosa Strain RBS Isolated from a Saltern, Tunisia: Effect of Metal Ion Cofactors on the Structure and the Activity of LasB
Source: Biomed Res Int. 2020 Jul 23;2020:6047528. doi: 10.1155/2020/6047528 (PMC7396000; doi:10.1155/2020/6047528)
Supplement: Supplementary Materials — Figure S1: casein hydrolysis. Figure S2: pyocyanin production by P. aeruginosa RBS on Pseudomonas agar P medium. Figure S3: SDS-PAGE and zymography assays of the bacterial lysate from P. aeruginosa strain RBS. Figure S4: initial velocity as a function of DQ-substrates (μg/mL). [file 6047528.f1.pdf]

Supplementary Materials

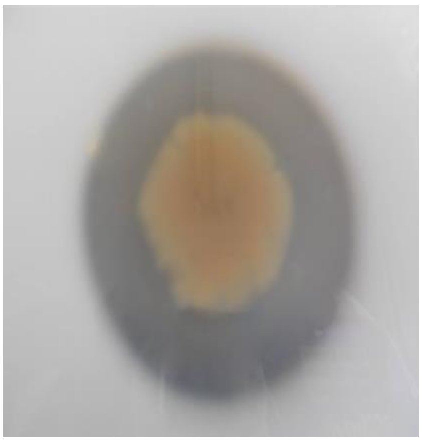

Figure S1: Casein hydrolysis zone of *P. aeruginosa* strain RBS on LB supplemented with casein (1%).

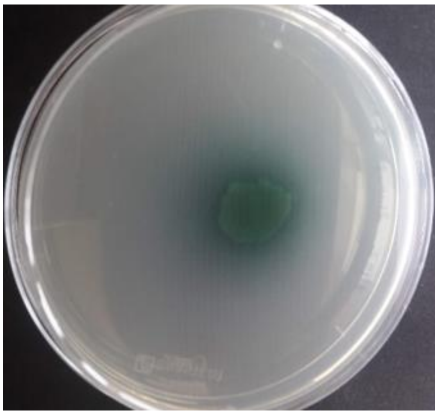

Figure S2: Pyocyanin production by *P. aeruginosa* RBS on Pseudomonas (ce n'est pas le nom d'une bactérie mais d'un produit, pas en italique) Agar P medium. The green pigment is attributed to pyocyanin production.

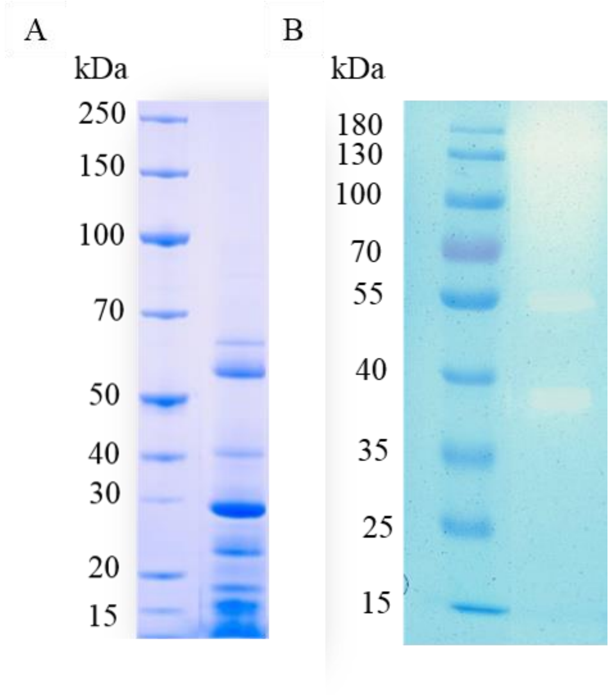

Figure S3 (A) SDS-PAGE analysis of a crude exract from *P. aeruginosa* strain RBS. Lane 1, PageRuler Broad-Range Protein Ladder (15 to 250 kDa). Lane 2, crude extract after ammonium sulfate fractionation. (B) Zymogram of a crude extract from *P. aeruginosa* strain RBS. Lane 1, PageRuler Prestained Protein Ladder (15 to 180 kDa). Lane 2, zymogramof the crude extract after ammonium sulfate fractionation.

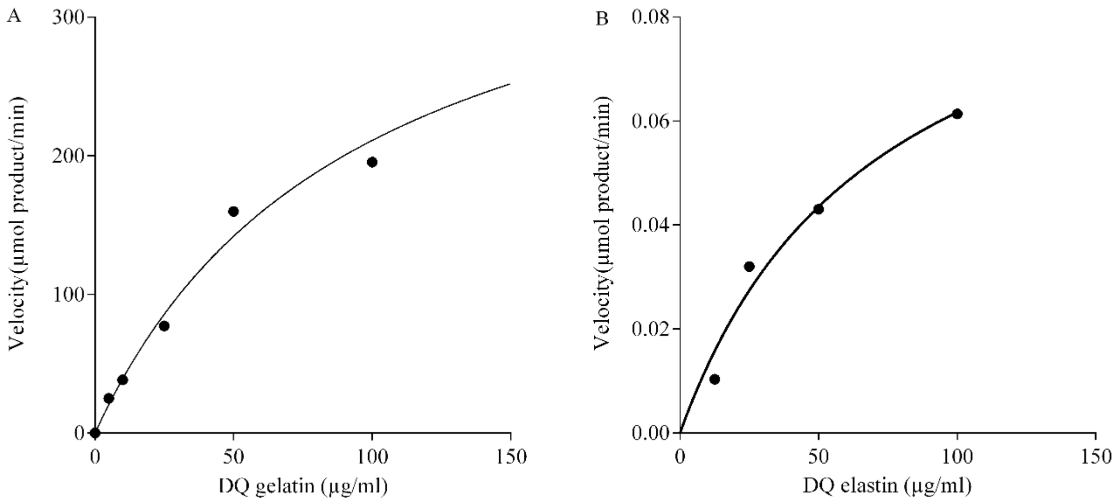

Figure S4: Initial velocity as a function of (A) DQ Gelatin and (B) DQ Elastin concentration (µg/mL). Activity assays were performed with 100 µg/mL of LasB. Prism 6 (GraphPad Software, Inc) was used to fit the data with the corresponding Michaelis-Menten curve and to calculate the V_{max} and K_m value.
